# Superb Microvascular Imaging Compared with Contrast-Enhanced Ultrasound for Assessing Laser Ablation Treatment of Benign Thyroid Nodules

**DOI:** 10.1155/2018/1025657

**Published:** 2018-12-23

**Authors:** Wengang Liu, Ping Zhou, Yongfeng Zhao, Shuangming Tian, Xiaomin Wu

**Affiliations:** Department of Ultrasound, The Third Xiangya Hospital of Central South University, Changsha, Hunan Province 410013, China

## Abstract

**Purpose:**

To compare superb microvascular imaging (SMI) with contrast-enhanced ultrasonography (CEUS) for evaluating the ablation of benign thyroid nodules.

**Methods:**

225 Patients with 256 benign thyroid nodules underwent conventional ultrasound, color Doppler flow imaging (CDFI), CEUS, and SMI before and after laser ablation. They were routinely followed up at 1, 3, 6, and 12 months. The volume and volume reduction rate of the ablated nodules was calculated.

**Results:**

On SMI, the complete ablated nodules had no microvascular perfusion, while the incompletely ablated nodules had microvascular perfusion at the edge of the nodule. The percentages of the detected incompletely ablated nodules of SMI (37/256, 14.45%) and CEUS (41/256, 16.02%) were comparable, and both were significantly higher than CDFI (*P*< 0.001). CEUS was used as the criterion to determine whether the nodules were completely ablated. The sensitivity, specificity, and accuracy of SMI for detecting incompletely ablated nodules were 90.2, 98.2, and 100%, respectively. The volume of ablated nodules, as measured on ultrasound, was greater than that on CEUS or SMI (both* P*< 0.001), while CEUS and SMI were similar. The average volume reduction rate of nodules at 1, 3, 6, and 12 months was 40.25, 54.98, 76.83, and 95.43%, respectively.

**Conclusion:**

SMI sensitively detected the capillaries within residual thyroid nodules after laser ablation. The lesion size and detection rate of incompletely ablated nodules via SMI was consistent with that of CEUS. SMI may replace CEUS in certain cases for monitoring the curative effect of laser ablation for benign thyroid nodules.

## 1. Introduction

Thyroid nodules are common clinical diseases, most of which are benign, but some benign nodules require treatment because of compression symptoms, affecting aesthetics or anxiety of malignant transformation. Although surgical resection is the most common method for treating thyroid nodules, patients are often not willing to accept because of invasion and scarring of the neck. Therefore, nonsurgical and minimally invasive treatment modalities, such as ethanol ablation, percutaneous laser ablation (PLA), and radiofrequency ablation (RFA), have been used to treat thyroid nodules and yielding good results. In 2000, Pacella et al. [1] were the first to describe using PLA for the treatment of thyroid nodule. Subsequent research and clinical applications have shown that PLA is safe and effective [2–4]. Ideally, PLA of the thyroid nodule inactivates the cells and coagulates the tissue. Necrotic tissue is then endocytosed by the body's immune system, and the lesion shrinks and gradually disappears [5].

However, determining whether the ablated thyroid nodule is completely necrotized is a problem, as both conventional and color Doppler ultrasound are limited for this purpose. To determine the curative effect of PLA, the microcirculation before and after ablation may be compared via contrast-enhanced ultrasonography (CEUS), reflected as changes in perfusion of the contrast agent. If the lesion is completely ablated, there is no contrast agent perfusion. The efficacy of CEUS for evaluating the completeness of ablation is similar to that of contrast-enhanced computed tomography (CECT) and magnetic resonance imaging (MRI) [6, 7]. However, while CEUS is preferred by researchers for evaluating the ablation effect [8, 9], clinically it still has disadvantages, such as adverse reactions and high cost.

A new modality for evaluating microvascular perfusion is superb microvascular imaging (SMI), a noninvasive ultrasonic technology. SMI has great sensitivity for detecting capillaries, which allows better differentiation between slow blood-velocity flow signals and movement artifacts. SMI has been applied for microvascular detection of thyroid, breast, and liver lesions [10–13] and reportedly can display microvasculature better than traditional color Doppler flow imaging (CDFI) and power Doppler imaging (PDI) [14].

Compared with conventional Doppler, SMI shows more accurately the microvascular distribution of thyroid nodules, and its sensitivity for microvascular detection of tissue is superior to that of CEUS [15]. Yet, there is little research concerning SMI for the evaluation of the curative effect of PLA of thyroid nodules. This study evaluated the value of SMI, relative to that of CEUS, for assessing the treatment outcome of laser ablation for benign thyroid nodules.

## 2. Methods

The Medical Ethics Committee of our hospital approved this retrospective study. The requirement for informed consent was waived.

### 2.1. Patients

This study retrospectively analyzed pathologically proven benign thyroid nodules treated with PLA under the guidance of ultrasound from June 2013 to December 2015 at Third Xiangya Hospital of Central South University.

For inclusion in this analysis, patients conformed to the following criteria: thyroid nodule with at least one diameter > 20 mm; neck compression symptoms, including neck discomfort and dysphagia; being with aesthetic requirements but rejected, or were unsuitable, for surgical resection; benign cytological findings (repeated at least twice); and normal thyroid function. Normal thyroid function was considered values within normal range of serum thyroid stimulating hormone (TSH), T3, free triiodothyronine 3 (FT3), T4, and free triiodothyronine 4 (FT4). Patients with any of the following were excluded from this study: coagulation disorders; pathological examination results that indicated malignant nodules; ultrasound findings which showed suspicious malignant nodules, e.g., microcalcification, irregular margins, or anteroposterior-to-transverse diameter ratio (A/T) > 1.

The study population comprised 225 patients (154 women, 71 men; aged 38 ± 19 y, range 18-63 y) with 256 benign thyroid nodules, in which 194 patients had a single nodule and 31 patients had multiple nodules. Multiple nodules in a patient were all located at the same thyroid lobe and were all ablated during the same procedure. 

### 2.2. PLA Method

After consultation regarding the PLA method, process, postoperative considerations, and the possibility of complications, all the patients provided preoperative informed consent for the PLA operation. PLA was performed with an Esaote EchoLaser integrated ultrasonic diagnosis and laser ablation system (EcholaserX4, Elesta, Florence, Italy). The machine was a MyLab Twice (Esaote, Elesta, Florence, Italy), with an LA332 high-frequency linear array probe, and a frequency of 3.0-11.0 MHz. The laser therapeutic apparatus was an EchoLaser X4 laser treatment system (EcholaserX4, Elesta, Florence, Italy). The Nd:YAG (neodymium-doped yttrium aluminum garnet) laser wavelength was 1064 ± 10 nm, the maximum output power of each fiber was 7 W ± 20%, and the length of the fiber was 15 cm with a diameter of 300 *μ*m.

With the patient supine, the neck fully exposed, and after sterilizing and anaesthetizing the local skin, a 21G needle (Gallini, Mantova, Italy) was used to puncture to the center of the targeted nodule under the guidance of ultrasound. The optical fiber was identically positioned through the core needle, and the guided needle stepped back 5 mm so that the tip of the optical fiber contacted the tissue directly.

After setting the relevant parameters of the laser light source (wavelength 1064 nm, power 3 W) and opening the laser emission, the treatment was begun. The number of ablation fibers and total energy was determined based on the shape and size of the nodules. In most patients, a nodule <15 mL could be ablated by a single fiber with the pinpoint located in the geometric center of the nodule. A nodule ≥15 mL was generally ablated by more than 2 fibers simultaneously. When multiple fibers were needed, they were parallel and within 10 mm of each other, depending on the shape of the nodule. If the nodule was large, all pinpoints and optical fibers could be synchronously drawn back 10 mm to conduct a second ablation based on the original needle arrangement, when necessary.

The entire treatment procedure was monitored by real-time ultrasound. As the energy is released from the laser light source, an irregular hyperechoic gasification area forms at the tip of the optical fiber, which expands. The treatment was ended when this area completely covered and was larger than the targeted nodule.

### 2.3. Curative Effect Assessment

In this study, CDFI and SMI were first performed for the patients one hour after ablation, followed by CEUS, and the curative effect of ablation was evaluated. As the postoperative pathological results were unavailable in this study, whether the nodule was completely ablated was determined based on CEUS, as follows. A completely ablated nodule was defined as a nodule without enhancement and with no contrast agent filling, and its range was larger than the original size of the nodule. An incompletely ablated nodule was identified as a small amount of residual tissue with irregular contrast agent perfusion along the margins of the nodule.

### 2.4. SMI Protocol

Prior to CEUS, SMI was performed by a sonographer with >5 years' experience. A Toshiba Aplio 500 ultrasonic diagnostic instrument (Toshiba Medical Systems, Tokyo, Japan) was used, with a 14L5 linear array probe and a frequency 14 MHz. SMI included both color and monochrome SMI, and the monochrome (grayscale) mode has improved sensitivity by subtracting the background information and focuses only on the vasculature. The probe was placed above the lesion and the sampling range was adjusted to cover the entire lesion and surrounding thyroid parenchyma. The color-gain was broadened as far as possible to avoid noise. Microvascular distribution within the ablated lesion was observed using a low-speed scale (1.0-2.0 cm/s) and it was thus determined whether there was residual tissue. The size of the area without blood flow on SMI was measured, and the volume of this region (V_1_) was calculated based on the following formula: V_1_ = *π*·*ghi* / 6, where* g*,* h*, and* i* were the length, width, and height, respectively.

### 2.5. CEUS Protocol

CEUS was performed after SMI by the same sonographer, using a Siemens Acuson S2000 ultrasonic diagnostic instrument (Siemens, Mountain View, CA, USA), with a 9L4 linear array probe and a frequency of 9 MHz. The size of the ablated nodule was measured on 2D ultrasonic images. The volume (V) of the target nodule was calculated as: V = *π*·*abc*/6, where* a*,* b*, and* c* were the length, width, and height of the ablated nodule, respectively.

The dynamic double contrast-imaging model was adopted with contrast-tuned imaging. The mechanical index was 0.03. The contrast agent was the second generation of SonoVue (Bracco, Milan, Italy), i.e., sulfur hexafluoride microbubbles enclosed by phospholipids. Twenty-five milligrams of SonoVue was diluted with 5 mL saline (0.9% Na Cl), and after vibrating for 30 s, a microbubble suspension was created. The suspension was injected as a bolus through the antecubital vein with a 20 G trocar; 2.4 mL for each injection was then flushed with 5 mL saline. The dynamic perfusion of the lesion was continuously observed for 90 s, and the images were stored.

Whether there was residual tissue was determined by the enhanced signal within the ablated lesion. The size of the nonenhanced area on CEUS was measured, and the volume (V_2_) was calculated as V_2_ = *π*·*def*/6, where* d*,* e*, and* f* were the length, width, and height of the area, respectively.

### 2.6. Imaging Interpretation

All 2D-ultrasound, CDFI, SMI, and CEUS images were independently analyzed by 2 sonographers with >5 years' experience with thyroid ultrasound diagnosis. If they had different opinions, they reviewed the images together until a consensus was reached.

Incompletely ablated nodules were determined based on the following criteria: on CDFI, blood flow signals present at the margins of nodules; on SMI, residual tissues with microvascular perfusions were at the margins of nodules; and on CEUS, a few residual tissues with irregular contrast agent perfusions were present at the margins of nodules.

### 2.7. Follow-Up

All patients were routinely followed up at 1, 3, 6, and 12 months after ablations. CDFI, SMI, and CEUS were performed to assess whether there was residual tissue or recurrence. The size and blood supply of the nodule was carefully observed. The volume of the nodule was calculated. The volume reduction rate (VRR) was calculated as VRR = [(preoperative volume – volume at follow-up) / preoperative volume] × 100%. The thyroid function was tested before and one month after ablation.

### 2.8. Statistical Analysis

All statistical analyses were performed using SPSS for Windows software (version 19.0, SPSS, Chicago, Ill, USA). All values are shown as mean ± standard deviation. Comparisons of the volume of the ablated nodule among the CDFI, CEUS, and SMI images were performed using the paired* t*-test. The rates of detected incomplete ablated nodules among the CDFI, CEUS, and SMI images were compared using McNemar's test. Calculations were performed for the sensitivity, specificity, and accuracy of SMI for diagnosing the accuracy of detecting incomplete ablation nodules. One-way analysis of variance was used to compare changes in nodule volume and VRR. Statistical significance was defined as* P *< 0.05.

## 3. Results

In this analysis, 225 patients (256 benign thyroid nodules) conformed to the study criteria (Table 1). All the patients were successfully treated with laser ablation. There were no injuries to the recurrent laryngeal nerve, trachea, or esophagus, and no neck vascular injuries or skin burns.

### 3.1. Preoperative and Postoperative Images of Thyroid Nodules

**Figure 1:**
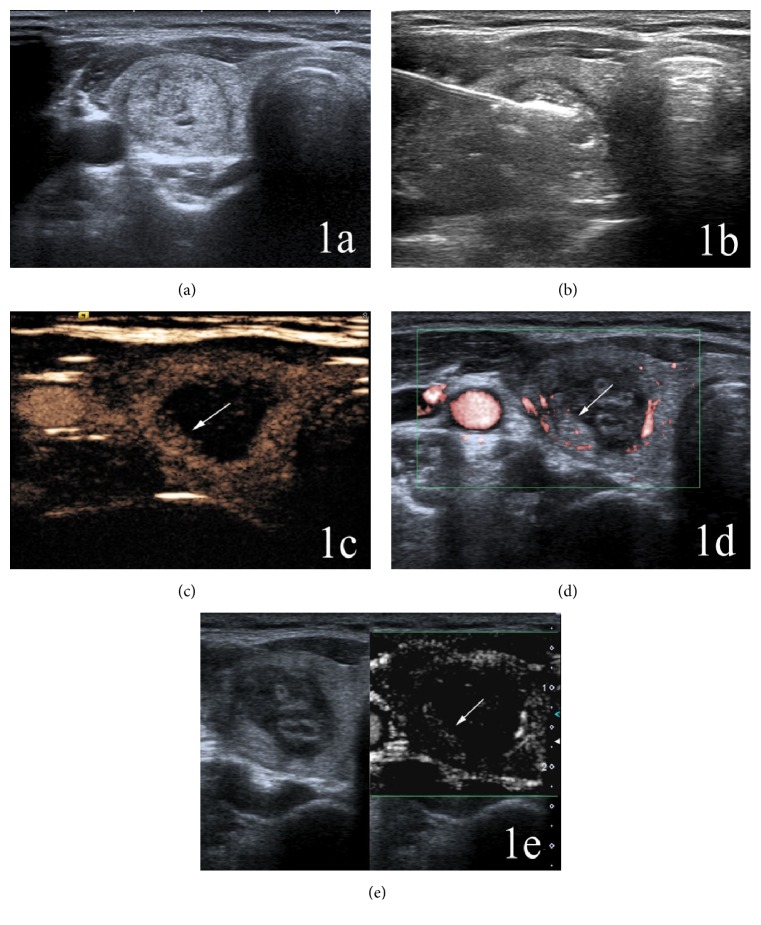
A 46-year-old woman with an incompletely ablated nodule in the right lobe of the thyroid. (a) The original size of the nodule was 23 mm × 14 mm × 16 mm. (b) Intraoperative 2D-ultrasound monitoring during laser ablation showed gasification at the front end of the optical fiber and a hypoechoic area at the periphery of the nodule. (c) Postoperative CEUS showed an enhanced signal (arrow) within the ablated lesion. (d) Color SMI revealed microvasculature (arrow) within the ablated lesion. (e) Monochrome SMI also showed microvasculature (arrow) within the ablated nodule, which had better contrast compared with the color SMI.

**Figure 2:**
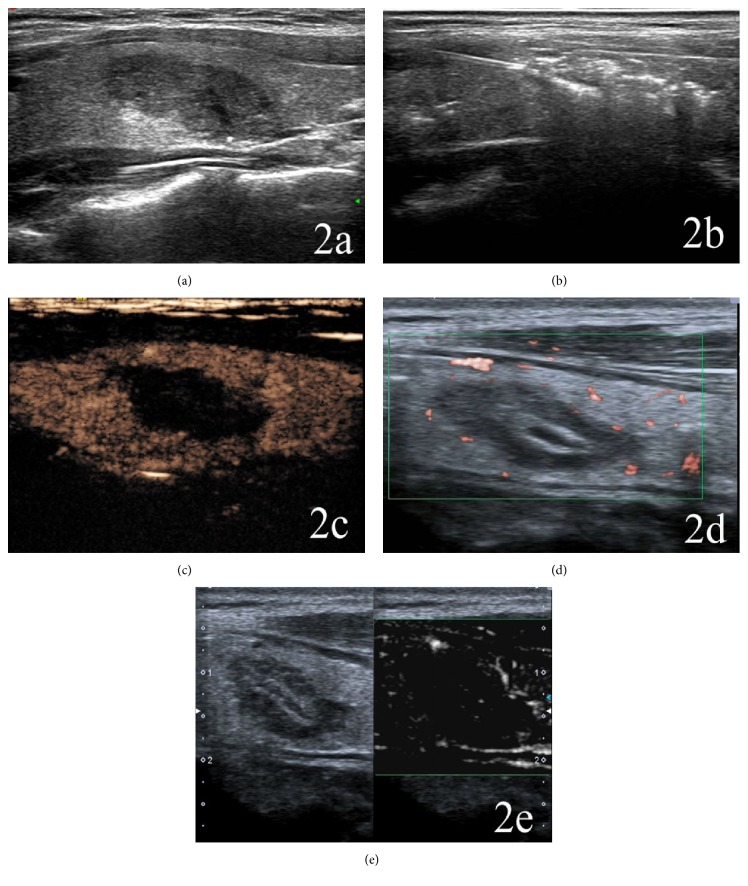
A 32-year-old man with a completely ablated nodule at the left lobe of the thyroid. (a) The original size of the nodule was 21 mm × 11 mm × 13 mm. (b) Intraoperative 2D-ultrasound monitoring showed that the nodule was completely covered by a patchy hyperecho gasification area. (c) Postoperative CEUS showed that no contrast agent filled the ablated region, which was not enhanced. Its range was larger than the original size of the nodule. (d) Color SMI showed that there was no microvascular perfusion within the ablated lesion. (e) Monochrome SMI also showed no microvascular perfusion within the ablation zone.

On CDFI, there were color blood flow signals within all nodules before ablation. The color Doppler signals within completely ablated nodules were absent after PLA, while blood flow signals were still present at the margins of some incompletely ablated nodules (Figures 1 and 2).

On CEUS, prior to PLA there were contrast agent perfusions within all nodules. After PLA, there were no contrast agent fillings and no enhancements within completely ablated nodules, and the regions of nodules were larger than the original sizes (Figures 1 and 2). There were a few residual tissues with irregular contrast agent perfusions at the margins of incompletely ablated nodules.

On SMI, before PLA, there were microvascular perfusions within all nodules. After PLA, there were no microvascular perfusions within completely ablated nodules, and the regions of nodules were larger than the original sizes. In incompletely ablated nodules there were residual tissues with microvascular perfusions (Figures 1 and 2).

### 3.2. Detection Rates for Incompletely Ablated Nodules among CDFI, CEUS, and SMI

Of the 256 nodules, 10, 37, and 41 were detected as incompletely ablated on CDFI, SMI, and CEUS, respectively (3.90%, 14.45%, and 16.02%). The difference in detection rates between the SMI and CEUS was not significant (*P* = 0.481), but both rates were significantly higher than that of CDFI (*P*< 0.001). In this study, CEUS was used as the criterion to determine whether the nodules were completely ablated. The sensitivity, specificity, and accuracy of SMI for detecting incomplete ablation nodules were 90.2%, 98.2%, and 100%, respectively. Supplementary ablations were performed for 41 incompletely ablated nodules 24 hours after the first ablations.

### 3.3. Postablation Volumes on CDFI, CEUS, and SMI

The postoperative volumes of ablated nodules were 15.10 ± 5.25mL on 2D-ultrasound, 12.15 ± 3.83 mL on CEUS, and 12.98 ± 4.34 mL on SMI. The mean volume of ablated nodules as measured on 2D-ultrasound was significantly greater than that of CEUS or SMI (both* P*< 0.001). The mean volume of ablated nodules on CEUS and SMI was comparable (*P* = 0.533).

### 3.4. Changes in Nodule Volume and VRR

As measured on 2D-ultrasound, the postoperative volume of nodules differed significantly from that of the preoperative volumes (all* P*< 0.001, Table 2). The average VRR of nodules at 1, 3, 6, and 12 months after ablation was 40.25, 54.98, 76.83, and 95.43%, respectively. The reduction in volumes over time was significant (*P*< 0.001).

## 4. Discussion

In this study, the completeness of ablation of benign thyroid nodules was evaluated and compared between SMI and CEUS. On SMI, microvascular perfusion was absent within completely ablated nodules, but present in incompletely ablated nodules at the margins of residual tissues. The rates of detection of incompletely ablated nodules using SMI and CEUS were comparable, and both of these were significantly higher than that of CDFI. When adopting CEUS as the criterion to determine whether the nodules were completely ablated, the accuracy, sensitivity, and specificity of using SMI for evaluating complete ablation was 100%, 90.2%, and 98.2%, respectively.

CDFI is not accurate for depicting the blood flow within the nodule, as there may be significant artifacts due to the heating effects and tissue gasification immediately after the ablation. In addition, CDFI employs a wall filter to eliminate noise and motion artifacts, which can easily cause loss of sensitivity for showing low-velocity blood flow in microvessels [16].

The ablation is ended when the transient hyperechoic area, produced by gasification of the tissue and cells exposed to heat, completely covers and exceeds the nodules [17, 18]. After this, the hyperechoic area more or less correlates with the area of coagulation necrosis, and can be considered a rough estimate of the extent of tumor necrosis. However, it does not accurately reflect the ablation area, and there may still exist residual tissue within the hypoechoic area after ablation [19]. As the contrast agent fills the target area of ablation, tiny residual lesions can be discovered on CEUS, indicating whether the lesion was completely ablated [20, 21]. Thus, CEUS can dynamically depict the vascular filling defect of the ablation area during PLA of the thyroid nodule, effectively monitoring the efficiency of the ablation and timing its conclusion. The diagnostic efficiency of CEUS for assessing the efficacy of ablation is reportedly comparable to that of CECT and MRI [7, 22].

Although CEUS is a preferred method for evaluating the effect of ablation, it is invasive and requires administration of contrast agent. Compared with traditional Doppler ultrasound, SMI technology can more accurately display greater details of microvascular flow and vessel branching in the peripheral and internal microvasculature of thyroid nodules, by identifying noises associated with blood flow and tissue movement and using an adaptive calculation method [23]. SMI simultaneously displays blood flow information as a 2D gray-scale and color Doppler, and the gray-scale pattern highlights blood flow information by suppressing 2D tissue information. Both the gray-scale and color modes depict microvasculature and low-velocity blood flow without contrast agents and truly reflect the perfusion status of the nodule [24, 25].

The new SMI technology has relatively improved signal-to-noise ratio, and may be able to detect microvascular blood flow signals ≥ 0.1 mm/s [26]. Machado et al. [10] preliminarily studied the microvascular distribution within the thyroid nodule with SMI and compared the results with CDFI and PDI. They found SMI could display more details of the capillaries within nodules and detect more microvascular blood flow signals [10]. Since SMI has potential value for detecting the microvasculature within the ablated lesion after ablation, in the present study we determined the efficiency of SMI for evaluating the ablation effect of thyroid nodules.

In the present study, SMI failed to detect four incompletely ablated nodules that were shown on CEUS. Studies have shown that CEUS can show capillaries with a diameter < 40 *μ*m and blood flow of ~1 mm/s [27]. The sensitivity of SMI for detection of blood flow within capillaries is less than that of CEUS. Therefore, CEUS can more clearly show tissue perfusion and the margins of a lesion that was not completely ablated. However, SMI has higher resolution and frame frequency compared with CEUS and can clearly show the capillary branches within incompletely ablated nodules. Importantly, SMI is more convenient and noninvasive in a clinical setting for monitoring ablated lesions, without using the contrast agent.

The present study found that the volume of ablated lesions revealed by 2D-ultrasound was greater than the volume shown on CEUS or SMI. This is consistent with the conclusion of an experimental study with rabbit liver [28]. The reason is due to the cooling effect of blood flow. The ablation temperature near blood vessels is lower than in the avascular area, which can cause tissue residues surrounding residual capillaries [29]. In the present study, the margins of ablated lesions were clearly seen on both CEUS and SMI, and the mean volumes of the ablated lesions were similar. This suggests that SMI can be an important tool for assessing volume change and ablation range in ablated lesions.

This study is limited in several ways. First, we did not have available postoperative pathology results, which are the gold standard for determining the presence of residual nodules. Second, the contrast-enhanced and super microvascular images were performed with different ultrasound devices, which may have affected our results. Finally, we only evaluated the short-term efficacy of laser ablation, and a long-term follow-up is important for determining the presence of residual nodules.

In conclusion, SMI can sensitively detect the capillaries within residual thyroid nodules after laser ablation, and the rate of detection for incompletely ablated nodules and evaluation of lesion size was consistent with that of CEUS.

## Figures and Tables

**Table 1:** General information of patients and pathologies of all thyroid nodules *∗*.

		n (%)
Gender	Male	71 (31.56)
	Female	154 (68.44)

Age, y		38 ± 19 (18-63)

Nodule location	Left lobe	140 (54.69)
	Right lobe	116 (45.31)

Nodules per lobe	Single	194 (86.22)
	Multiple	31 (13.78)

Pathological pattern	Nodular goiter	215 (83.98)
	Adenoma	41 (16.02)

Ablation fibers		1. 32 ± 0.51(1~3)
Duration of ablation procedure, min		26.8 ± 11.2(16.2~54.0)
Gross energy, J		4825 ± 1875 (2916~9720)

*∗* Reported as n (%), unless indicated otherwise

**Table 2:** Changes in nodule volume and VRR.

	Pre-treatment	1st month	3rd month	6th month	12th month	*P* value
Nodules, n	256	234	223	228	211	—
Mean volume, mL	10.22 ± 5.63 (3.12~26.58)	6.11 ± 2.93 (2.08~21.45)	4.61 ± 2.13 (1.66~14.61)	2.37 ± 1.22 (0.40~8.60)	0.47 ± 0.33 (0.07~1.86)	<0.001
Largest diameter, cm	3.28 ± 1.55 (2.01~4.10)	2.74 ± 1.18 (1.62~3.83)	2.47 ± 0.99 (1.25~3.31)	1.93 ± 0.89 (0.75~2.33)	1.13 ± 0.56 (0.42~1.44)	0.002
VRR, %	—	40.25	54.98	76.83	95.45	<0.001

## Data Availability

The data used to support the findings of this study are available from the corresponding author upon request.
